# Computer-aided detection for radiological disease severity classification on chest radiograph in children with intra-thoracic tuberculosis

**DOI:** 10.1371/journal.pgph.0006547

**Published:** 2026-06-17

**Authors:** Megan Palmer, Ineke Derks, H. Simon Schaaf, Pierre Goussard, Devan Jaganath, Sandra V. Kik, Diana M. Gibb, Anna Turkova, Elisabetta Walters, Marieke M. van der Zalm, Anneke C. Hesseling, James A. Seddon

**Affiliations:** 1 Desmond Tutu TB Centre, Department of Paediatrics and Child Health, Faculty of Medicine and Health Sciences, Stellenbosch University, Cape Town, South Africa; 2 Paediatric Pulmonology, Department of Paediatrics and Child Health, Stellenbosch University, Cape Town, South Africa; 3 Division of Paediatric Infectious Diseases, University of California, San Francisco, United States of America; 4 TB Programme, FIND, Geneva, Switzerland; 5 Medical Research Council Clinical Trials Unit, University College London, London, United Kingdom; 6 Great North Children’s Hospital, Newcastle upon Tyne Hospitals Trust, Newcastle, United Kingdom; 7 Department of Infectious Disease, Imperial College London, London, United Kingdom; ICMR-National Institute for Research in Tuberculosis: National Institute of Research in Tuberculosis, INDIA

## Abstract

Implementation of the World Health Organization’s (WHO) recommended shorter 4-month treatment regimen for non-severe tuberculosis (TB) in children requires classification of disease severity on chest X-ray (CXR). Access to specialists for CXR interpretation is limited. We explored the use of computer-aided detection of CXR (“CAD”) to automate CXR classification of radiological disease severity. To do this, we combined three CXR datasets from children with confirmed and clinically diagnosed TB across the disease spectrum. CXRs were independently classified as radiologically severe or non-severe by two expert human readers. Definition of radiological disease severity aligned with WHO guidelines. CAD scores were generated by CAD4TB v7.0 and qXR v3.0 software. Neither software product was specifically trained with paediatric CXRs or for disease severity classification. We compared CAD scores between CXRs classified by human readers as non-severe versus CXRs classified by human readers as severe. CXRs from 526 children were included in this analysis: median age was 2.1 years (inter-quartile range 1-4.2 years); 57% of the children had microbiologically confirmed TB. We found that median CAD scores were significantly lower for CXRs classified as non-severe versus severe by human readers; the difference was greatest in children >5 years. The area under the receiver operating curve was 0.82 and 0.78 for qXR, and 0.79 and 0.76 for CAD4TB, against the reference of ‘severe’ as classified by each individual human reader respectively. These results demonstrate that CAD is a promising tool for TB disease severity stratification and has the potential to support access to shorter TB treatment regimens for children. Investment in paediatric CAD training and development to optimize solutions for children beyond the TB screening and diagnosis use-case is warranted.

## Author summary

This manuscript explores the performance of computer-aided detection (“CAD”) software to differentiate between radiologically severe and non-severe disease on chest X-rays from children with tuberculosis. With additional paediatric-specific optimization, CAD could be a useful tool for disease severity stratification in children.

## Background

Tuberculosis (TB) is both preventable and treatable, yet the disease remains a leading cause of death in low- and middle-income countries. Its eradication is a global health priority [1,2]. A key strategy towards TB control is the optimization of treatment regimens, including treatment shortening [3]. Shorter treatment regimens have been proven as effective, are more acceptable to patients, with fewer cumulative side effects and are more cost-effective for TB programmes [4–8].

In 2022 the World Health Organization (WHO), based on the results from the multi-country, randomized SHINE TB treatment-shortening trial, made the recommendation that children with non-severe drug-susceptible TB could be treated for 4 months instead of 6 months, with the same TB drugs given at the same doses [6,9,10]. In these guidelines, and in SHINE, the classification of ‘non-severe disease’ was largely based on clinician interpretation of the chest x-ray (CXR) [9]. Although treatment shortening was classified as a ‘strong recommendation’ in these WHO guidelines and could be implemented without TB programmes needing to procure novel TB drugs or adjust dosing guidelines, only five countries had implemented this recommendation by the end of 2023 [1,11]. An obstacle to implementation is limited access to CXR and to experienced clinicians to interpret paediatric CXRs for radiological disease severity classification.

CXR interpretation by humans is hampered by variable inter-reader agreement and suboptimal accuracy [12–14]. For identifying the radiological features of pneumonia on paediatric CXRs, the range of inter-reader agreement is wide, with reported kappa coefficients between 0.14 and 0.62 [15–18]. More specifically related to CXR features of TB in children, agreement on the presence of peri-hilar lymphadenopathy ranges from none to substantial (kappa -0.01-0.79), with similar ranges reported for agreement between readers to identify airway compression (kappa -0.04 to 0.79) [13,19–21]. In the treatment-shortening context, an additional challenge is that radiological classification of disease severity is a new skill that requires training, re-training and ongoing evaluation [22]. While this capacity building is important and ongoing, other innovative approaches to support disease severity stratification and facilitate faster uptake and implementation of treatment shortening guidelines warrant exploration.

Computer-aided detection of CXRs (CAD), not yet recommended for use in children <15 years by the WHO, offers the possibility of an immediate standardized CXR interpretation score, overcoming the limitation of variable inter-reader agreement. With accumulating data from adults demonstrating that CAD could outperform (or be equivalent to) human readers in terms of accuracy in the context of TB screening and triage, CAD could provide immediate ‘expert equivalent’ CXR classification at the point-of-care, without the need for referral [23,24].

CAD development in the context of TB has been appropriately focused on the screening and triage use-case, but there are clear operational gains in CAD replacing human-read CXR at all decision points where CXR is currently used in the TB care pathway. This includes CXR for radiological disease severity classification to inform treatment-duration decisions. Hypothesising that radiologically severe TB disease on CXR will generate higher CAD scores than non-severe disease, we explored the association between disease severity on CXR, as classified by expert human readers, and CAD scores, in children with TB.

## Methods

### Ethics statement

The data included in this analysis was collected from children who were enrolled into three separate studies which were all approved by the Stellenbosch University Health Research Ethics Committee (SU HREC) - reference numbers M14/09/044, N11/09/28 and 2003/005 respectively.

### Description of clinical cohorts combined in the final dataset

For this analysis we combined datasets which included CXRs from three TB diagnostic or treatment studies carried out at Tygerberg Hospital in Cape Town, South Africa. These studies were selected to achieve a final merged dataset that represented an appropriate balance of CXRs across the disease severity spectrum. For children enrolled into Cohorts 1 and 2 (described below) written informed consent was obtained from legal guardians of all children (and assent from children >7 years of age, as appropriate) prior to enrollment. Consent was waived by the SU HREC for children enrolled into Cohort 3 which is a surveillance study. All CXRs were taken at a single radiology department. The classification of ‘microbiologically confirmed TB’ in children in all studies was based on ≥1 sample (respiratory or other) being positive on the Xpert MTB/RIF assay and/or positive for *Mycobacterium tuberculosis (Mtb)* on liquid culture. All children in all study cohorts had ≥ 1 respiratory sample collected for *Mtb* microbiological evaluation at the time of clinical presentation/enrolment. The following clinical variables were collected in all studies and collated in the final merged dataset: age, sex, HIV infection status, weight-for-age Z-score (WAZ) and *Mtb* microbiological confirmation status.

Cohort 1 included children <16 years enrolled in the SHINE treatment-shortening trial in South Africa between July 2016 and July 2018, with a diagnosis of TB classified as non-severe at enrollment. Detailed eligibility and trial data are described elsewhere [6].

Cohort 2 included children <12 years with TB disease who were enrolled with presumptive TB in a prospective TB diagnostic study between March 2012 and March 2017. Eligibility is described elsewhere [25]. Data from children diagnosed with TB at enrolment and retrospectively classified as having ‘confirmed’ or ‘unconfirmed’ TB as per standard research case definitions were included in this analysis [26].

Cohort 3 included children <12 years with microbiologically confirmed TB from a paediatric TB surveillance study enrolled between December 2011 and May 2014. Children were identified through laboratory surveillance of all Xpert MTB/RIF and *Mtb* culture results in routine care at Tygerberg Hospital. Demographic and clinical details were captured for each child [27].

### CXR classification methods

For each child, the baseline CXR image was retrieved for analysis. Each CXR (in jpeg or tif format) in the final merged dataset was reviewed by two expert human readers who first classified each CXR as technically acceptable or not. All CXRs were then classified as normal or abnormal and, if abnormal, as radiologically severe or non-severe. Lateral images, if available, were viewed by the human readers but not processed by the CAD software. Each of the two expert human readers classified each CXR independently. Radiological disease severity classification aligned with the WHO definitions, which were based on those used in the SHINE trial. In these definitions, non-severe disease is defined as consolidation (if present) confined to <1 lobe with no cavitary disease, no miliary infiltrates, no airway compression and no complicated pleural effusion (defined as loculated or associated with underlying consolidation) [6,28]. Normal CXRs were classified as non-severe.

Both human readers were paediatric specialists with >25 years clinical and research experience in the interpretation of CXRs for paediatric TB and specific experience in the radiological classification of disease severity for the SHINE trial. The readers were aware that the CXRs were from children diagnosed with TB but were blinded to all other clinical information and to each other’s interpretation.

### CAD methodology

CXRs classified as ‘technically unacceptable’ by ≥1 human reader were excluded from the CAD analysis. Output from two CAD software products were used in this analysis: qXR version 3.0 from Qure.ai and CAD4TB version 7.0 from Delft Imaging. Both products generate a TB score (0–100 for CAD4TB, 0–1 for qXR) based on certainty of radiological TB (with a higher score representing a higher probability of TB abnormalities being present). The analyses were conducted independently and were not focused on a comparison between software systems. Neither software product was specifically trained to assess radiological disease severity, and both products were predominantly trained using adult CXRs.

### Statistical Methods

**Fig 1:**
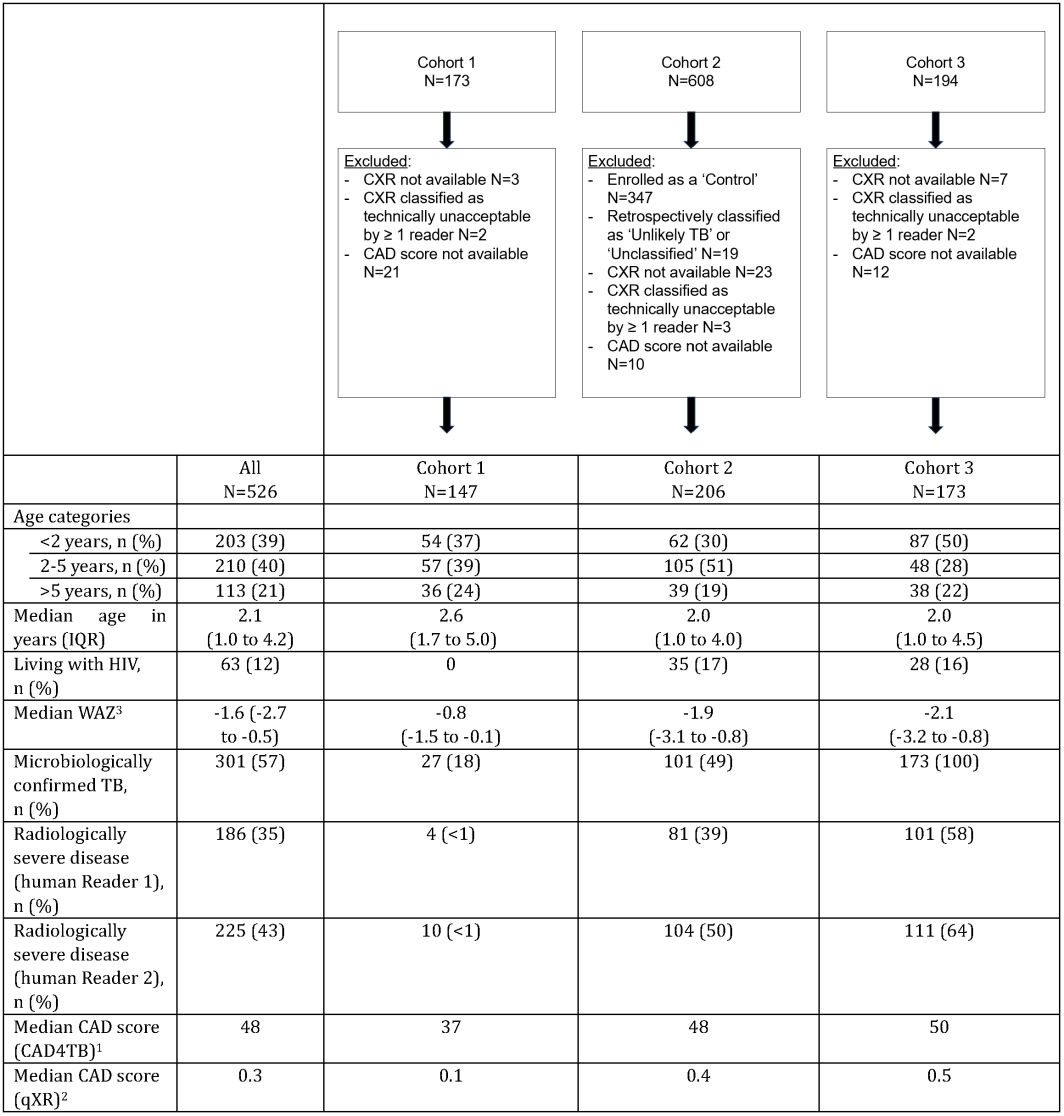
Description of the three cohorts included in the final dataset for this analysis. ^1^CAD4TB score range 0-100, ^2^Qure.ai score range 0-1 TB: tuberculosis; CXR: chest X-ray; CAD: Computer Aided Detection; IQR: Inter-Quartile Range; WAZ: weight-for-age Z-score.

Only complete records with a classification from both CXR readers and both software systems, were included in the final analysis. All statistical analyses were conducted using R Statistical Software [29]. Descriptive statistics were used to summarize the data (**Fig 1**).

Cohen’s Kappa statistic was used to assess the level of agreement between the two readers [30]. Agreement was defined as both readers classifying a CXR as severe or non-severe. The Landis & Koch scale was used to classify the magnitude of agreement for the Kappa coefficient: 0-0.20 = slight, 0.21-0.40 = fair, 0.41-0.60 = moderate, 0.61-0.80 = substantial, 0.81-1.00 = almost perfect [31]. The distribution of CAD scores was assessed for normality to determine whether parametric statistical tests were appropriate. The normality of the data was assessed using the Anderson-Darling test [32]. As the data violated the normality assumption, non-parametric tests were used. The Wilcoxon rank-sum test, also known as the Mann-Whitney U test, was used to test whether there was a statistically significant difference in scores between severe and non-severe readings [33]. These analyses were performed independently for each software system and human reader. To account for multiple comparisons across the software systems and human readers, p-values were adjusted using Holm-Bonferroni correction. Effect sizes were calculated to quantify the magnitude of differences, and an adjusted p-value less than 0.05 was considered statistically significant.

**Fig 2:**
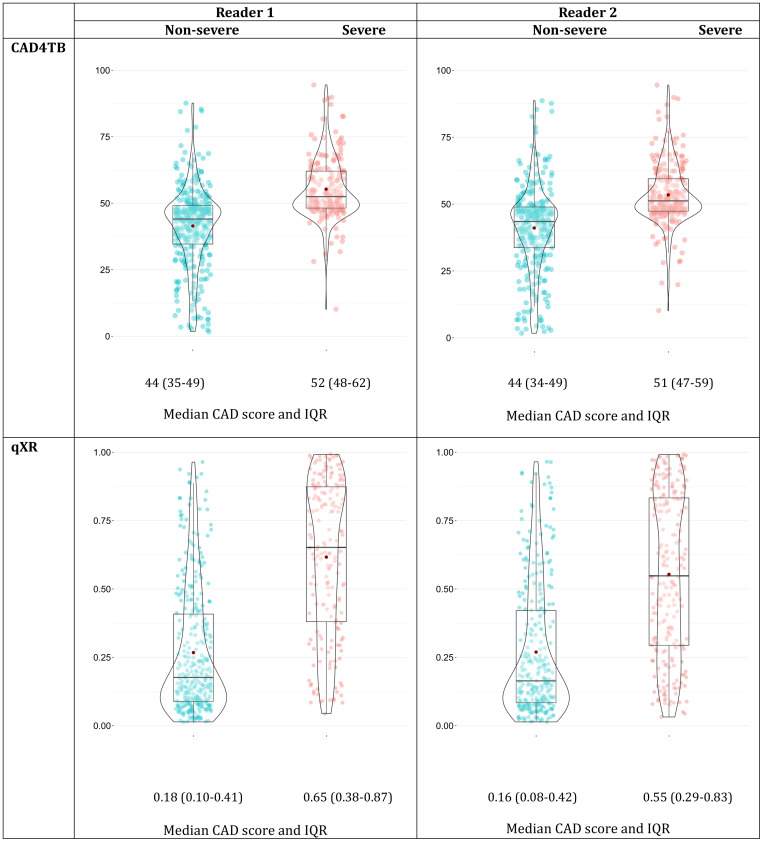
Violin plots representing distribution of CAD scores for CXRs classified as severe and non-severe by individual readers. In these figures the CAD score is plotted on the y-axis with range 0-100 (CAD4TB) and 0-1 (qXR) and the human classification of severity is represented as blue/green for non-severe and orange for severe. The red dot on each violin plot represents the mean CAD score. P-value <0.05 for all comparisons. CAD: Computer Aided Detection; IQR: Inter-Quartile Range.

**Fig 3:**
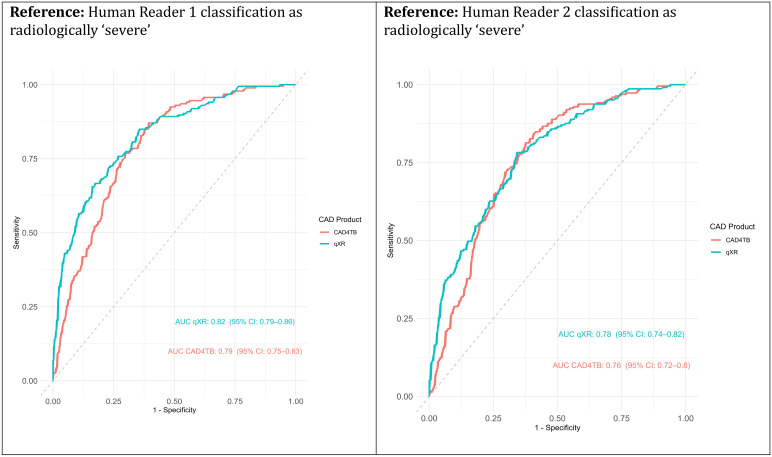
ROC curves representing CAD scores from CAD4TB v7.0 (orange) and qXR v3.0 (blue/green) against the reference of chest x-rays classified as radiologically severe by humans: Reader 1 (left) and Reader 2 (right). ROC: Receiver Operating Curve; CAD: Computer Aided Detection.

**Fig 4:**
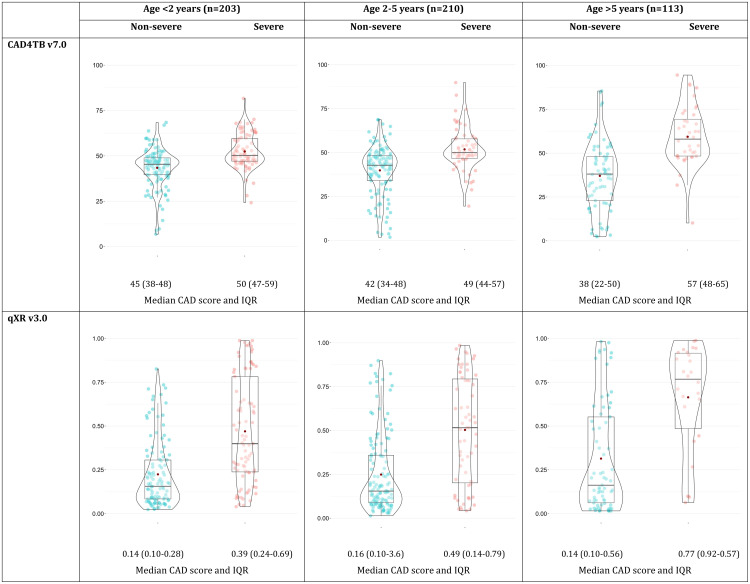
Violin plots representing distribution of CAD scores for CXRs classified as severe versus non-severe by human Reader 1^1^ by age category. In these figures the CAD score is plotted on the y-axis with range 0-100 (CAD4TB) and 0-1 (qXR) and the human classification of severity is represented as blue/green for non-severe and orange for severe. The red dot on each violin plot represents the mean CAD score. P-value <0.05 for all comparisons. ^1^Results by human Reader 2 available in **S1 Fig**. CAD: Computer Aided Detection; IQR: Inter-Quartile Range.

CAD scores generated from each software product were visualized using box-and-whisker plots with a violin plot overlay to illustrate the distribution and potential data outliers. These visualizations were stratified by reader, by radiological severity category overall (**Fig 2**) and by age categories (0–2 years, 2–5 years and >5 years) (**Fig 4;** **S1 Fig**). Receiver operating characteristics (ROC) curves were used to evaluate the performance of each software product to distinguish radiologically severe from non-severe disease as classified by human readers, and the area under the curve (AUC) was calculated (Fig 3).

**Fig 5:**
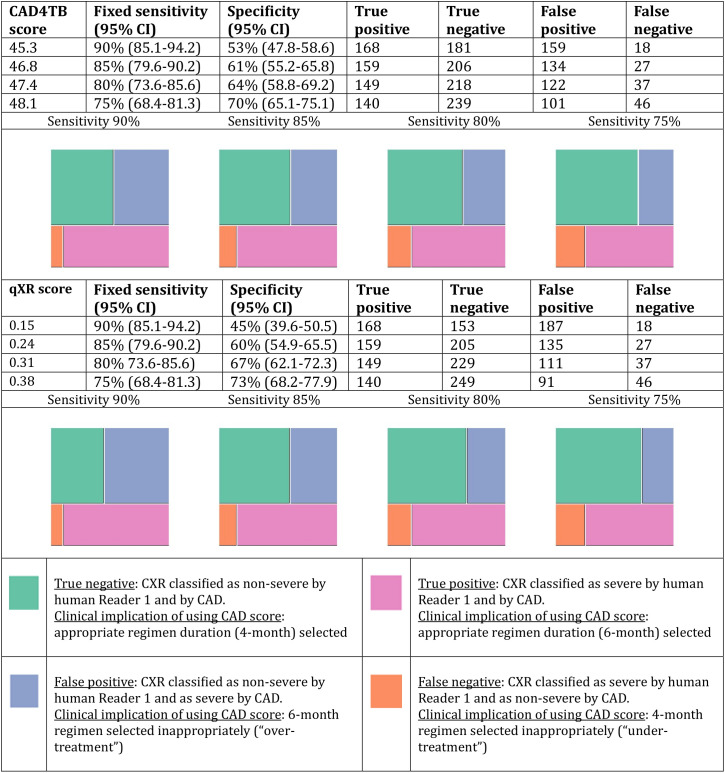
Performance of CAD software to correctly predict radiologically severe disease as classified by human Reader 1^1^ at different sensitivity and CAD thresholds in our combined cohort (N = 526, disease severity prevalence 35%). ^1^ Comparison to human Reader 2 included as **S2 Fig**. Mosaic plots generated in R Statistical Software [28].

Sensitivity and specificity were calculated by applying a range of threshold scores to binarize them as severe or non-severe. For each threshold, classification metrics were computed, and the thresholds yielding fixed sensitivity levels (90%, 85%, 80%, and 75%) were selected to report the corresponding specificity, including 95% confidence intervals, and to generate mosaic plots (**Fig 5**). This approach allows for the evaluation of CAD performance at different sensitivity thresholds which may be specifically relevant in different programmatic use-cases. This was repeated for a theoretical population of 1000 children with a lower prevalence of severe disease of 10%. (**S2 Fig**).

**Fig 6:**
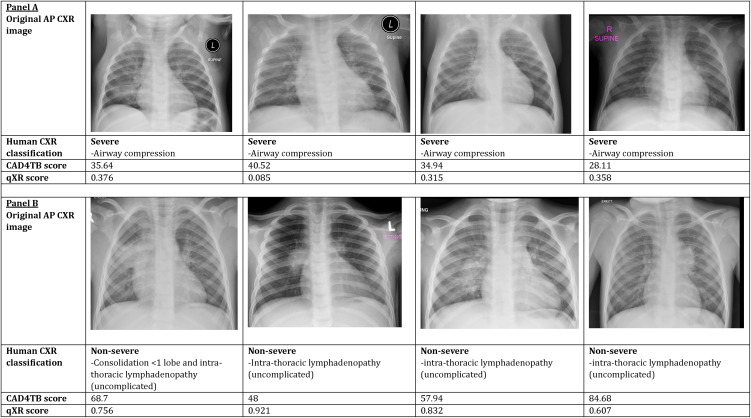
Illustrative CXR image sets classified by humans as severe but with low CAD scores and as non-severe but with high CAD scores. Panel A represents CXR images classified as ‘severe’ by human readers but with low CAD scores and Panel B represents CXRs classified as ‘non-severe’ by human readers but with high CAD scores. CAD: Computer Aided Detection; CXR: chest X-ray.

To explore which radiological patterns the CAD software may be ‘misclassifying’, two sets of CXR images were visually reviewed: CXRs which both human readers classified as ‘severe’ but had low CAD scores (<5^th^ percentile) from one/both software products, and CXRs which both human readers classified as ‘non-severe’ but had high CAD scores (>95^th^ percentile) for either or both software products. Eight of these CXRs are included in **Fig 6** to illustrate radiological features that may be causing discrepant classification between humans and CAD.

### Findings

The median age of the overall merged cohort of 526 participants was 2.1 years (IQR 1-4.2 years), 63/526 (12%) were living with HIV and 301/526 (57%) had microbiologically confirmed TB. Few CXRs (<1%) from children enrolled in Cohort 1 were classified as severe by human readers, while Cohort 3 had the highest proportion of severe CXRs (58% and 64% by Reader 1 and 2 respectively). Overall, Reader 1 classified 186/526 (35%) CXRs as severe, and Reader 2 classified 225/526 (43%) as severe. CAD scores from both software products were lowest in Cohort 1 (37 for CAD4TB and 0.1 for qXR) and highest for Cohort 3 (50 for CAD4TB and 0.5 for qXR) (**Fig 1**). Inter-reader agreement on radiological disease severity between the two human readers was substantial (kappa 0.62, 95% CI 0.56-0.69) [31].

The median CAD scores generated by both software products for CXRs classified as non-severe by either human reader were lower (44 and 0.18 by Reader 1; 44 and 0.16 by Reader 2 for CAD4TB and qXR respectively) than for CXRs classified as severe (52 and 0.65 by Reader 1; 51 and 0.55 by Reader 2). This difference in median CAD scores was statistically significant (p < 0.05) against both human readers and for CAD scores generated by both CAD software products (**Fig 2**). On visual interpretation of the violin plots, CAD4TB software performed better at identifying CXRs classified as severe (clustering towards higher CAD scores and very few with low CAD scores) and qXR software performed better at identifying those classified as non-severe (clustering towards lower CAD scores) (**Fig 2**). When compared against the reference of radiologically severe disease on CXR as classified by the human readers, the AUC for qXR was 0.82 (95% CI 0.79-0.86) and 0.78 (95% CI 0.74-0.82), and for CAD4TB was 0.79 (95% CI 0.75-0.83) and 0.76 (95% CI 0.72-0.80), against Reader 1 and Reader 2, respectively (**Fig 3**).

In a sub-group analysis by age, the difference in median CAD scores between CXRs classified as severe versus non-severe, increased in each ascending age group with a difference in scores of 5, 7 and 19 for CAD4TB and 0.25, 0.33 and 0.63 for qXR in the respective age groups of <2 years, 2–5 years and >5 years (**Fig 4**).

To represent the proportion of CXRs that the CAD software products would correctly and incorrectly classify (using human Reader 1 as the reference) we fixed their sensitivity at 90%, 85%, 80% and 75%. Optimising sensitivity at 90%, the corresponding CAD threshold and specificity for qXR was 0.15 and 45% and for CAD4TB it was 45.3 and 53%. At this fixed sensitivity qXR correctly classified 321/526 (61%) CXRs (168 true positives and 153 true negatives) and CAD4TB correctly classified 349/526 (66%) CXRs (168 true positives and 181 true negatives). The qXR and CAD4TB software mis-classified 187 (35%) and 159 (30%) CXRs respectively as representing severe disease that was classified as non-severe by human Reader 1. If applied in a real-world setting these children would receive the 6-month treatment regimen when they could have benefited from the 4-month regimen. Inversely, both software products mis-classified 18 (3%) CXRs as non-severe that were classified by human Reader 1 as severe. These children would potentially be ‘under-treated’ by receiving the shortened 4-month treatment regimen. (**Fig 5**).

In the theoretical cohort of 1000 children with lower TB disease severity prevalence (10%) (**S2 Fig**), at a fixed sensitivity of 90%, 42% and 50% of children would be ‘over-treated’ using qXR and CAD4TB classifications respectively, and 1% of children ‘under-treated’.

On visual review of the CXR image sets with discordance between human classification of severity and CAD scores (**Fig 6**), a notable finding from the CXRs classified as ‘severe’ by human readers but with lower CAD scores (**Fig 6, Panel A**), was the presence of airway compression. In the CXR image set classified as ‘non-severe’ by humans but with higher CAD scores (**Fig 6, Panel B**), several CXRs showed large intra-thoracic lymph nodes but minimal parenchymal involvement and no airway compression (uncomplicated lymph node disease).

### Interpretation

In this study we found a difference in CAD scores between CXRs from children diagnosed with TB and classified as radiologically severe by human readers compared to those classified as radiologically non-severe. This finding was consistent for both of the CAD software products that we evaluated and despite the fact that neither product was previously trained for the disease severity use-case. This difference was larger in older children, an unsurprising result, given that the software products were largely trained on adult data and the anatomy and radiological features of TB on CXR are more similar between adults and older children than between adults and younger children.

While our results are encouraging and support further investigation of CAD for the disease severity stratification use-case, CAD performance was not optimal. Software training with paediatric CXR images and for this specific use-case is required to optimize model performance given that the radiological features of TB disease, and severe TB disease specifically, differ between young children (especially those <5 years) and adults. A key feature of severe disease on CXR in young children is the narrowing or deviation of the large airways, a result of external compression by enlarged intra-thoracic lymph nodes which is a feature not often seen in adult TB. We did not systematically evaluate CAD performance against specific radiological patterns classified by humans, but on visual inspection of the set of discordant images, we found that airway compression was a common radiological features in the subset of CXRs classified as severe by humans but with low CAD scores. Evaluation of the shape and calibre of the large airways on CXR should be a focus area of CAD development in the context of paediatric TB generally, and disease severity stratification more specifically.

Although there is no comparative data from other paediatric studies, evidence from adult populations supports the potential of CAD as a disease severity prediction tool with machine learning models for severity predictions being successfully developed on CXR datasets for COVID19 [34–36]. Chandra et al developed a conventional machine-learning model to predict severity defined as a ratio of normal to abnormal lung parenchyma with a high degree of agreement (correlation coefficient r = 0.96) between the model and the expert radiologist’s severity score, while Sahoo et al successfully developed a severity score based on a percentage of ‘infected pixels’ trained against a standard severity score that estimated the proportion of infected lung on CXR [25,26]. This learning is encouraging, but the radiological manifestations of TB are heterogenous, and severity scores based solely on the proportion of lung parenchyma involvement will likely underperform in predicting severe TB disease. Specifically focused on TB, Kantipudi et al evaluated three deep-learning approaches to predict the Timika score, a CXR scoring system specifically developed for predicting TB severity in adults [37]. The correlation of their best-performing model with the Timika score was good (r = 0.70-0.84) and was based on the proportion of abnormal lung involved as well as the presence of cavities.

Our study has several strengths. The dataset included CXRs from a large total cohort of children with a high proportion of microbiologically confirmed TB (57%) and a wide disease spectrum (35–43% of CXRs were classified as ‘severe’ by human readers). This provides a high degree of certainty that these children truly had TB disease and also ensures that the full paediatric disease severity spectrum was represented in the CXR dataset. The clinicians who interpreted the CXRs to provide the reference human reads for this analysis have specific experience and expertise in the field of paediatric TB and radiological disease severity classification, providing a sufficiently robust benchmark against which to evaluate CAD performance. Poor inter-reader agreement is a well-recognised challenge of CXR interpretation and can limit confidence in study findings. However, the inter-reader agreement for the classification of severe versus non-severe between our two readers was classified as substantial, better than published inter-reader agreement for other TB CXR features [12,13].

Combining three CXR datasets taken from the same institution for this analysis allowed selection of CXRs across the full disease severity spectrum and reduced potential confounders such as differing CXR acquisition techniques and systematic variations in CXR quality. However, it does limit the generalizability of the results to other settings, especially primary care settings where the prevalence of severe radiological disease is expected to be lower. Our exploration of the performance of these CAD products in a theoretical lower TB disease prevalence setting (**S1 Fig**, **S2 Fig**) improves the generalizability of our findings to some degree.

An additional limitation, and a persistent challenge related to analyses that benchmark CAD performance against CXR interpretation by human readers, is the absence of a “ground truth” (gold standard). We acknowledge that training CAD and subsequent evaluation against an advanced imaging modality, such as computed tomography (CT) of the chest, would be the optimal strategy to determine whether CAD can outperform human-interpreted CXR. However, access to advanced imaging remains unfeasible in most research settings for use in young children, given the high costs, radiation exposure and need for sedation for image acquisition. Decision-making around disease severity and treatment duration in most high-TB burden countries is currently made by non-specialist clinicians at primary healthcare level. Therefore, if CAD performance can reach equivalence to CXR interpretation by an experienced specialist clinician then it will be a useful tool to support clinical decisions, even if CAD is not optimized against an imaging gold standard such as chest CT.

CAD software output of a ‘probability’ score rather than a binary classification of ‘severe’ versus ‘non-severe’ more closely reflects the pathophysiological process of TB disease progression and, arguably, may ultimately offer a more accurate approximation of true radiological severity across the disease spectrum. CAD score outputs also lend themselves to integration within score-based decision-making algorithms, such as the WHO’s Treatment Decision Algorithm, and severity prediction models. These approaches have particular value in paediatrics given the absence of a good diagnostic test and clinician comfort with the use of clinical algorithms for TB diagnosis.

Our findings, from a combined dataset from young children, provide an important contribution towards closing evidence gaps around the use of CAD in children and support further development of CAD as a tool for disease severity stratification to facilitate access to WHO-recommended shorter treatment regimens for paediatric TB. We argue that, given the existing reliance on CXR to make decisions around TB diagnosis and management in young children, this group has the most to gain from the automation of CXR interpretation and disease severity stratification systems. Ongoing and future investment is warranted to optimize CAD solutions for children, including the extension to multiple use-cases within the paediatric TB management pathway.

## Supporting information

S1 Fig Violin plots representing distribution of CAD scores for CXRs classified as severe and non-severe by human Reader 2^1^ by age category. **In these figures the CAD score is plotted on the y-axis with range 0–100 (CAD4TB) and 0–1 (qXR) and the human classification of severity is represented as blue for non-severe and pink for severe. The red dot on each violin plot represents the mean CAD score. P-value <0.05 for all comparisons.** ^1^Classification by Reader 1 included in main results Fig 4.
(TIF)

S2 Fig Performance of CAD software to correctly predict radiologically severe disease using Reader 1 classification of severity at different sensitivity and CAD thresholds in a theoretical cohort with lower disease severity prevalence (N = 1000, disease severity prevalence 10%). Mosaic plots generated in R Statistical Software [28].
(TIF)
