# Supplementary figures and images for: Computer-aided detection for radiological disease severity classification on chest radiograph in children with intra-thoracic tuberculosis
Source: PLOS Glob Public Health. 2026 Jun 17;6(6):e0006547. doi: 10.1371/journal.pgph.0006547 (PMC13274902; doi:10.1371/journal.pgph.0006547)

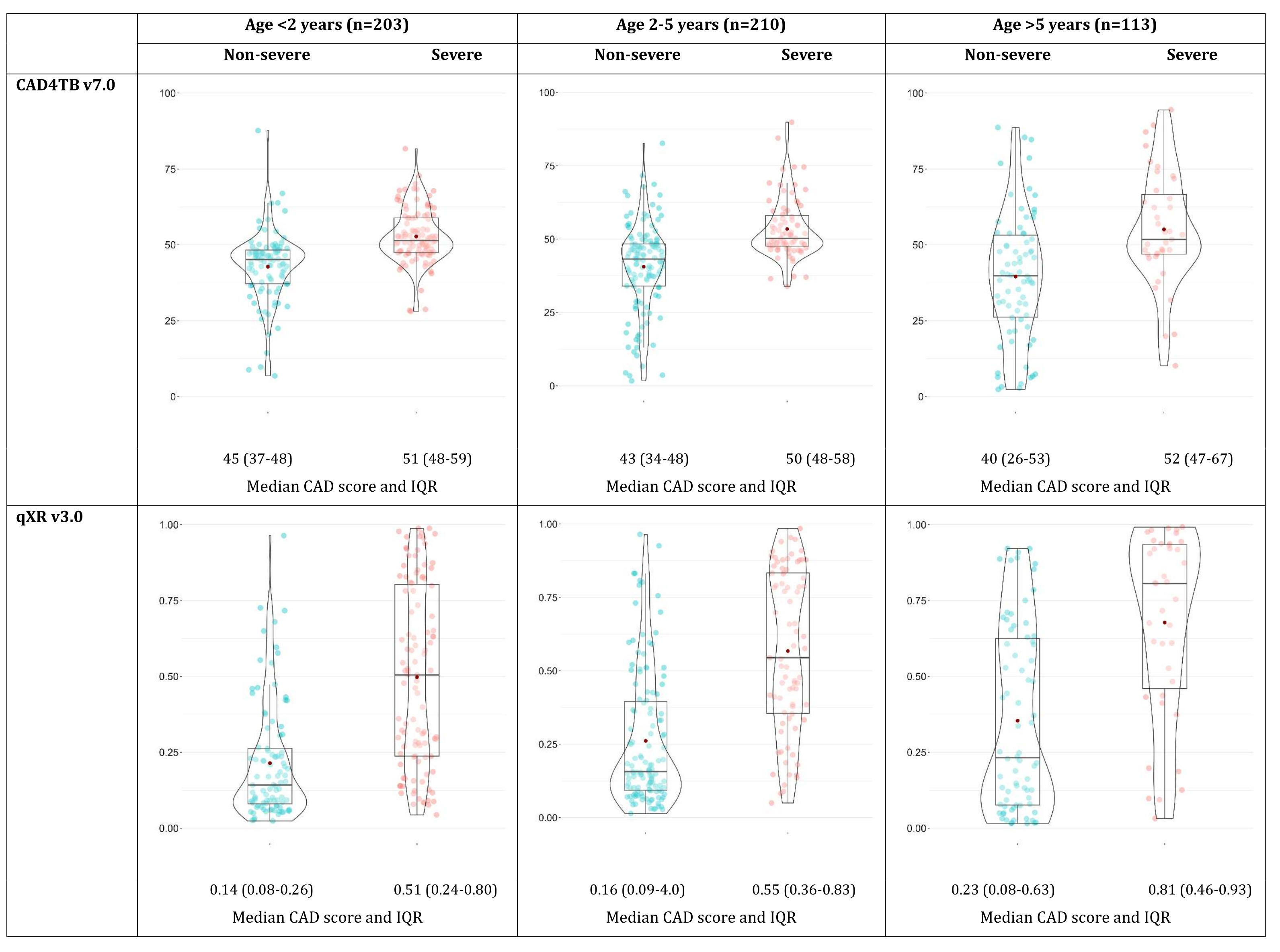

Supplement: S1 Fig — In these figures the CAD score is plotted on the y-axis with range 0–100 (CAD4TB) and 0–1 (qXR) and the human classification of severity is represented as blue for non-severe and pink for severe. The red dot on each violin plot represents the mean CAD score. P-value <0.05 for all comparisons. 1Classification by Reader 1 included in main results Fig 4. (TIF) [file pgph.0006547.s001.tif]

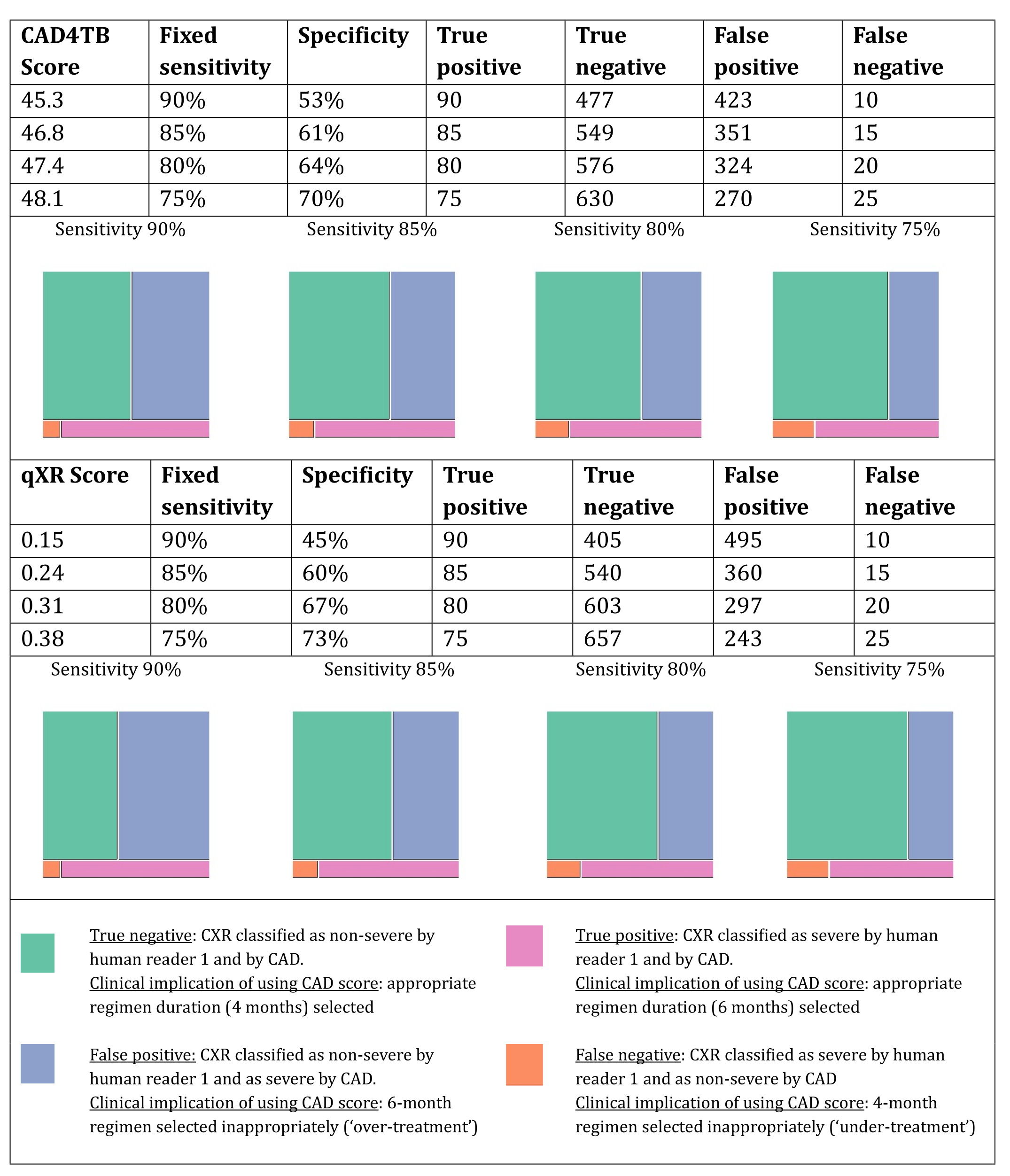

Supplement: S2 Fig — Mosaic plots generated in R Statistical Software [28]. (TIF) [file pgph.0006547.s002.tif]
